# Authors' reply

**DOI:** 10.1055/s-0042-1757970

**Published:** 2022-12-29

**Authors:** Carla Dinamerica Kobayashi, Victor Bertollo Gomes Porto, Martha Elizabeth Brasil da Nóbrega, Cibelle Mendes Cabral, Tiago Dahrug Barros, Cecília Maria Roteli Martins

**Affiliations:** 1 Coordenação Geral do Programa Nacional de Imunizações, Ministério da Saúde, Brasília, DF, Brazil; 2 Centro Universitário Faculdade de Medicina do ABC, Santo André, SP, Brazil

Adverse Events Related to COVID-19 Vaccines in Pregnant Women: Correspondence

Spontaneous abortion is the most frequent adverse outcome in pregnancy, with a rate of 10% to 20% of all pregnancies. In Brazil, the rate of abortion before the COVID-19 pandemic was of 3.5%. In our study, we observed 24 miscarriages after 678,025 doses of COVID-19 vaccines had been administered in pregnant women in Brazil, which corresponds to an incidence of 3.5 cases per 100,000 doses. 1 Such incidence is lower than the postvaccination rate of abortion related to COVID-19 vaccines described by Trostle et al. 2 and much lower than the historical 3.5% expected rate in Brazil.

Our study was observational and used passive surveillance data. Such studies cannot describe the exact rates of abortion in vaccinated pregnant women. Nonetheless, given the historical rates in Brazil, the expected number of abortions in 678,025 normal pregnancies would be 23,731, which averages out to 616 abortions per week of observation. Even if we consider reporting rates lower than 5%, our incidence of abortions would still be far below the expected. 3

Currently, the Brazilian Ministry of Health and the Pan American Health Organization (PAHO) are conducting a prospective active surveillance study with women who were vaccinated against COVID-19 during pregnancy in the 5 regions of the country. Such study will enable a more accurate description of the true rates of abortion and other adverse pregnancy outcomes in vaccinated women.

It is already known that pregnant women have a higher risk of developing severe COVID-19 and an increased risk of having adverse maternal and neonatal outcomes after COVID-19. We believe that our data, in association with the previously-published literature, suggests that the benefits of vaccinating pregnant women against COVID-19 far outweighs the risk of the disease. 4
